# 2405: Coping strategies used by caregivers of newly diagnosed pediatric brain tumor patients

**DOI:** 10.1017/cts.2017.271

**Published:** 2018-05-10

**Authors:** Alexandra Cutillo, Susan Davies, Avi Madan-Swain, Wendy Landier, Anastasia Arynchyna, Brandon Rocque

## Abstract

OBJECTIVES/SPECIFIC AIMS: The goal of this study is to use patient-centered qualitative techniques to determine what strategies caregivers use to cope with the stress of a child having recently (ie, within the past month) undergone surgical removal of a brain tumor. Results will eventually be evaluated and compared with results of quantitative measures of psychosocial risk and distress as well as demographic and medical characteristics. METHODS/STUDY POPULATION: All caregivers of patients with a newly diagnosed brain tumor requiring neurosurgery admitted to Children’s of Alabama (with English or Spanish-speaking parents) are eligible for enrollment. Participants are enrolled during their child’s initial hospitalization for surgical removal of a brain tumor. Approximately 1 month after hospital discharge, during a routine follow-up clinic visit, caregivers participate in a semistructured interview with a research assistant. Interview questions are used to obtain information about parent and family coping by asking first broadly about stress management over the previous month and then specifically about individual coping strategies. Semistructured interviews are audio recorded, transcribed, and coded for common themes. Interviews are coded by using specific words or phrases to describe various domains of the experience from the caregiver’s perspective. Each participant is given a study ID and study IDs are logged with each code word or phrase endorsed during the interview. RESULTS/ANTICIPATED RESULTS: To date, 22 caregivers have been enrolled and 15 have completed interviews. The most common coping mechanisms fall into the domains of active, avoidance, emotion-focused, and spiritual coping. Active coping consists of information seeking (eg, taking notes, internet research, asking questions), openly communicating emotions, celebrating small victories (eg, focusing on a good scan or test result, thinking that the diagnosis or treatment could have been worse), planning (eg, focusing on 1 d at a time), and maintaining normalcy (eg, maintaining extracurricular activities, returning to school if possible, continuing to see family and friends). Avoidance coping consists of evading discussions about emotions, withdrawal from family members, denial (eg, keeping a cancer diagnosis from the child), and avoiding seeing people or participating in activities. Emotion-focused coping consists of crying, laughing, and staying strong in front of the patient. In general, those who self-identify as coping poorly tend to be those who utilized more avoidance-focused coping strategies. Further, caregivers tended to identify active coping strategies (eg, taking notes, focusing on 1 appointment or treatment at a time) as the most helpful. DISCUSSION/SIGNIFICANCE OF IMPACT: It will be helpful for providers to more deeply understand the experience of caregivers whose children have recently undergone brain tumor resection and the strategies used to cope with the stress of the first month postsurgery. This information can be used to create standardized interventions for use during posthospitalization clinic visits. For example, if families continue to endorse that active coping mechanisms are the most helpful, providers can assist caregivers in developing these strategies (eg, uniformly provide notebooks and encourage caregivers to keep track of questions and appointment information, pair caregivers who are struggling with others who use more active coping strategies). Those utilizing more avoidance coping strategies may need more coaching and recommendations. A brief assessment could potentially be developed for caregivers dealing with this diagnosis, in order to quickly assess coping strategies and provide appropriate recommendations. Future analyses will determine whether initial coping strategies and adjustment are predicted by child age or medical information.

